# A New β-Galactosidase from the Antarctic Bacterium *Alteromonas* sp. ANT48 and Its Potential in Formation of Prebiotic Galacto-Oligosaccharides

**DOI:** 10.3390/md17110599

**Published:** 2019-10-23

**Authors:** Shangyong Li, Xiangjie Zhu, Mengxin Xing

**Affiliations:** 1 Department of Pharmacology, School of Basic Medicine, Qingdao University, Qingdao 266071, China; lisy@qdu.edu.cn; 2 Yellow Sea Fisheries Research Institute, Chinese Academy of Fishery Sciences, Qingdao 266071, China; zhuxiangjie1204@163.com

**Keywords:** β-galactosidase, galactooligosaccharides, daily industry

## Abstract

As an important medical enzyme, β-galactosidases catalyze transgalactosylation to form prebiotic Galacto-Oligosaccharides (GOS) that assist in improving the effect of intestinal flora on human health. In this study, a new glycoside hydrolase family 2 (GH2) β-galactosidase-encoding gene, *galA*, was cloned from the Antarctic bacterium *Alteromonas* sp. ANT48 and expressed in *Escherichia coli.* The recombinant β-galactosidase GalA was optimal at pH 7.0 and stable at pH 6.6–7.0, which are conditions suitable for the dairy environment. Meanwhile, GalA showed most activity at 50 °C and retained more than 80% of its initial activity below 40 °C, which makes this enzyme stable in normal conditions. Molecular docking with lactose suggested that GalA could efficiently recognize and catalyze lactose substrates. Furthermore, GalA efficiently catalyzed lactose degradation and transgalactosylation of GOS in milk. A total of 90.6% of the lactose in milk could be hydrolyzed within 15 min at 40 °C, and the GOS yield reached 30.9%. These properties make GalA a good candidate for further applications.

## 1. Introduction

β-Galactosidases (EC 3.2.1.23) are a type of glycoside hydrolase (GH), catalyzing lactose hydrolysis and transgalactosylation. As a commonly used medical enzyme, β-galactosidase has been widely used to decompose lactose into galactose and glucose in the dairy industry [1]. In addition, β-galactosidase can also produce Galacto-Oligosaccharides (GOS) via transglycosylation during the hydrolysis of lactose or other structurally related galactosides [2,3]. Hence, β-galactosidases can be widely used in applications related to nutrition and food processing [1]. 

To date, β-galactosidases have been purified and characterized from various organisms, such as bacteria [4,5,6,7], fungi [8,9], yeast [10,11], plants [12], and mammals [13]. Based on functional similarities, β-galactosidases can be classified into four GH families (GH 1, 2, 35, and 42) in the CAZy database [14,15]. Structurally, the most extensively studied β-galactosidase is lacZ from *Escherichia coli* that belongs to GH family 2 [16]. It is basically a tetramer of four identical amino acid chains, each of which consists of five domains. The third domain is an eight-stranded α/β barrel (TIM-type barrel), which acts as the active site made up of two different tetramer subunits [1,17]. 

The hydrolytic activity of β-galactosidase has been utilized in the food industry for decades to assist in absorbing undigested lactose [2]. Currently, many children and adults worldwide, especially in East Asia, are facing the problem of lactose dyspepsia and intolerance caused by lactase deficiency. [13,18,19]. The decreased activity or reduced synthesis of β-galactosidase, which occurs in the small intestine, is the reason behind these disorders [1,13]. About 2%−5% of infants within the first one to three months of life suffer from cow milk-protein intolerance. Approximately 70% of the world’s adult population cannot digest lactose, of which more than 90% are East Asians [20]. People who suffer from lactose intolerance only consume dairy-fermented products that comprise either no or very little lactose [21]. For overcoming the limitations imposed by lactose intolerance, β-galactosidases derived from bacteria and fungi can be used to make milk almost lactose-free by degrading the lactose in milk, which is sweeter than regular milk [13,18]. In addition, people take medicines containing β-galactosidase before consuming milk products, which can also effectively reduce lactose concentration [19,20]. Furthermore, in the food industry, the hydrolytic activity of β-galactosidases is also utilized for reducing crystallization in ice creams and condensed milk [22].

The transgalactosylation activity of β-galactosidase has also attracted considerable attention in recent years for the production of GOS, nondigestible prebiotics that assist in improving the effect of intestinal flora on human health, and stimulating the growth of beneficial bacteria, such as bifidobacteria and lactobacilli [1]. It is well known that the growth of probiotics can reduce the number of pathogenic bacteria, improve immunity, and prevent cancer [3]. Some research exhibited how gut microbiota or potential probiotics can exert therapeutic interventions by decreasing gut permeability or stimulating host homeostasis of the mucosal immune system and then regulating microbiota dysbiosis to a healthy state [23]. And in another study, information of microbial disorder-related inflammatory bowel disease was added and demonstrated how Rifaximin and Mutaflor exhibited synergic anti-inflammatory and therapeutic effects on acetic acid-induced colitis in rats [24]. Moreover, some synthetic bifunctional salts provide better biological control of pathogenic bacteria and thus serve as a therapeutic strategy for some microbial infections or disorders [25]. Additionally, GOS has various health benefits for the human body, such as decreasing the pH of human feces, preventing cariogenicity, and reducing serum cholesterol levels [1,2]. Therefore, in the food industry, GOS are used as an ingredient in infant milk and cereal-based food, soft drinks, and low-calorie sweeteners. At present, commercial GOS is produced via enzymatic synthesis (β-galactosidases) for good stereoselectivity and regioselectivity [26].

Thermostable activity is indispensable for the practical application of enzymes, as it reduces the risk of contamination, increases reaction velocities, decreases product inhibition, prolongs half-lives, and improves the solubility of substrates [4,26,27]. In this study, a new β-galactosidase from *Alteromonas* sp. ANT48, a strain isolated from the surface seawater of the Antarctic, has been purified and characterized. Subsequently, the enzyme was used to catalyze the transglycosylation reaction to produce oligosaccharides, for evaluating the potential application in GOS biosynthesis.

## 2. Results

### 2.1. Cloning and Sequence Analysis of GalA 

The marine bacterium *Alteromonas* sp. ANT48 was isolated from the surface seawater of Drake Passage near the Great Wall Station in the Antarctic. The 16S rDNA of *Alteromonas* sp. ANT48 showed high homology (99.63%) with *Alteromonas* sp. NJSX39 (accession number EF061431). The β-galactosidase gene, *galA*, from *Alteromonas* sp. ANT48, contained an intact open reading frame (ORF) of 3120 bp and encoded a protein consisting of 1039 amino acids. The theoretical isoelectric point (pI) and molecular weight (Mw) of β-galactosidase GalA were 5.27 and 117.40 kDa, respectively. According to a search of the Carbohydrate-Active enZymes (CAZy) database, conservative domain database in NCBI (CDD), and Pfam database, GalA was considered a new β-galactosidase of the glycoside hydrolase (GH) family 2.

**Figure 1:**
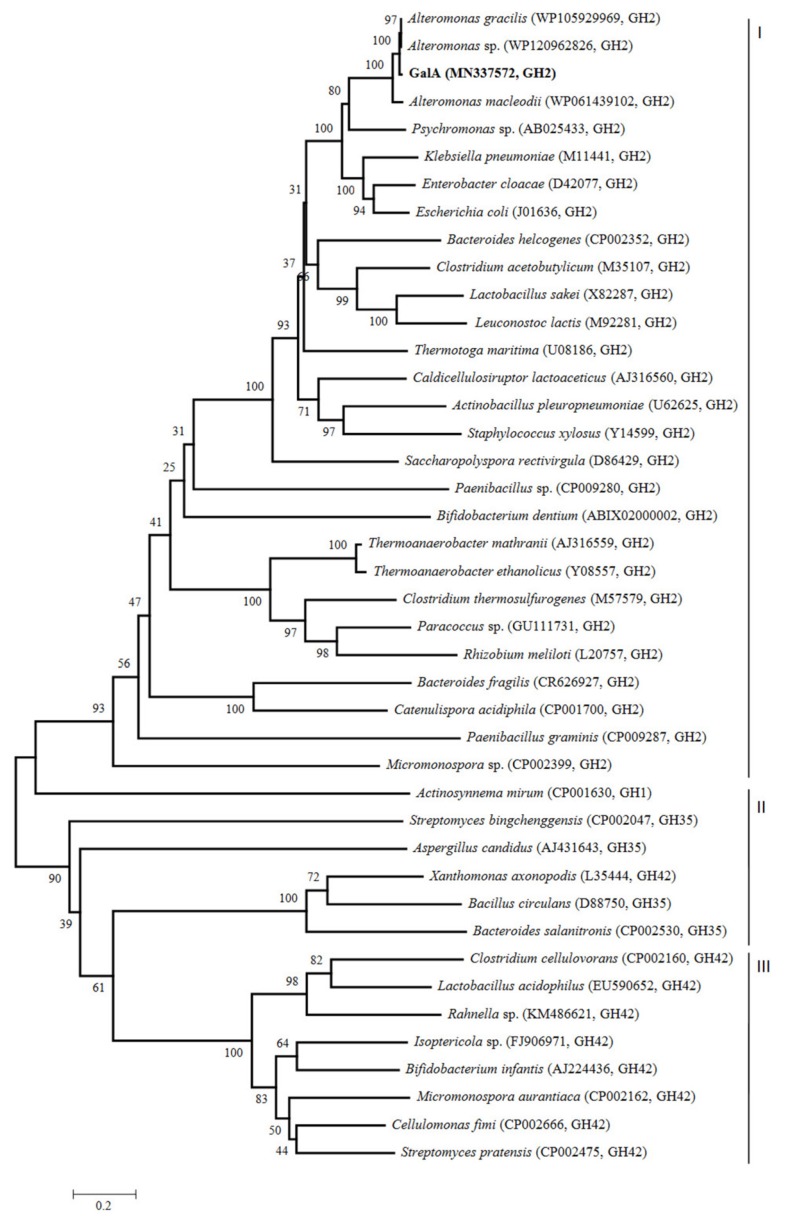
Evolutionary tree of β-galactosidases amino acid sequences based on the neighbor-joining method. The phylogenetic tree (1000 bootstraps) was constructed using the MEGA 6.0 program.

A phylogenetic tree was created based on the amino acid sequences of GalA and other reported β-galactosidases from GH1, GH2, GH35, and GH42 families (Figure 1). As expected, the resulting tree showed that GalA belonged to a group of enzymes designated as GH family 2 (shown as group I in Figure 1), while other β-galactosidases belonged to the group of enzymes classified as GH family 1 or GH35 (shown as group II in Figure 1) and the GH42 family (shown as group III in Figure 1). In the cluster of GH2, GalA showed highest identity with other β-galactosidases from the genus *Alteromonas*. In addition, GalA also showed closer phylogenetic relationship with β-galactosidases from *Psychromonas* sp., *Klebsiella pneumonia*, *Enterobacter cloacae*, and *Escherichia coli*, exhibiting 52%, 47%, 48%, and 49% identities, respectively.

**Figure 2:**
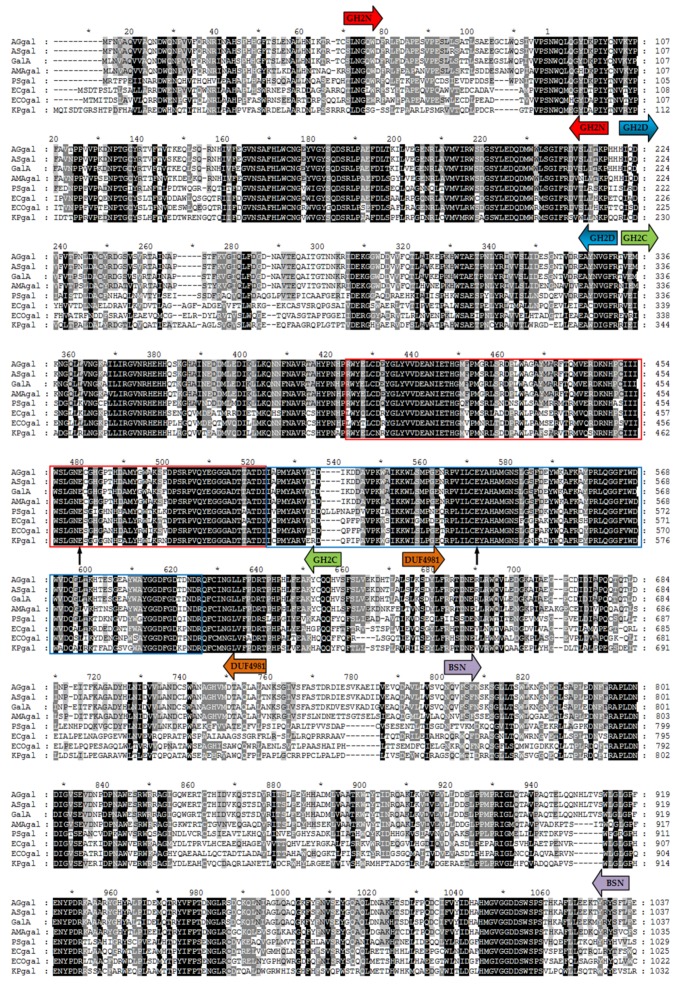
Comparison of the sequence of GalA with those of related β-galactosidases from GH2: AGgal from *A. gracilis* (WP105929969), ASgal from *Alteromonas* sp. (WP120962826), AMAgal from *A. macleodii* (WP061439102), PSgal from *Psychromonas* sp. (AB025433), ECgal from *E. cloacae* (D42077), ECOgal from *E. coli* (J01636), and KPgal from *K. pneumoniae* (M11441). The acid-base active sites and the consensus nucleophilic region are indicated by red and blue bands, respectively. The catalytic amino acids are marked by the black arrows. The domains of the GH2 are indicated by arrows of different colors.

Multiple alignments of the amino acid sequences were used to further analyze the conserved and catalytic domains (Figure 2). According to the Pfam database, three conserved structural domains occurred in GalA, consisting of the glyco hydro 2 N domain (GH2N, sugar binding domain, amino acid (aa) 47-aa218), glyco hydro 2 domain (GH2D, aa220-aa332), and glyco hydro 2 C domain (GH2C, TIM barrel domain, aa334-aa628). Among them, GH2C was the only domain related to the catalytic activity of GalA, which contained acid-base active sites and the conserved nucleophilic region with a high similarity to the typical β-galactosidases of the GH family 2, such as in *E. coli*. The proposed active site glutamic acid residues (GLU-460 and GLU-536) were also indicated after comparing with β-galactosidases from *E. coli*. However, the two other structural domains of GalA, domain of unknown function (DUF4981, aa639-aa726) and the Bgal small N domain (BSN, aa760-aa1036), showed low homology with other β-galactosidases from different bacteria. The results described above identified GalA as a new member of the GH family 2. 

According to the NCBI database, the sequences of β-galactosidase from the *Alteromonas* genus were fewer than those from other bacteria, and studies on the isolation and characterization of this enzyme from the *Alteromonas* genus were also limited. In this study, a new β-galactosidase from *Alteromonas* sp. ANT48, GalA, was purified and characterized. Sequence analysis showed that GalA belonged to the GH family 2 with five structural domains, namely GH2N, GH2D, GH2C, DUF4981, and BSN domains. Talens-Perales et al. structurally divided the GH2 into five groups [28]. According to this classification, GalA was classified as DA (domain architecture) type 3 with Bgal small N domain (BSN), linked to GH2C via a β-sandwich domain, while DA type 4 and 5 lacked the BSN domain. Previous studies have demonstrated that DA type 3 exhibited high transgalactosylation activity and synthesized β-(1,3) and β-(1,6) galacto-oligosaccharides [28,29], while DA type 5 synthesized β-(1,4) GOS [30,31]. Furthermore, using multiple alignments and docking analysis, both Glu460 and Glu536 in the GH2C (TIM barrel domain) of GalA were identified as catalytic residues; the consensus nucleophilic regions were also detected, which confirmed that the TIM barrel domain contained the catalytic sites and most of them participated in the formation of the active pocket [28]. Further docking analysis demonstrated that the highly hydrophilic semi-open pocket was on the catalytic domain of GalA, which is in agreement with the results of a previous study showing that the structure of the TIM barrel domain was suitable for hydrolyzing polysaccharides by modifying the catalytic conformation from cleft-shaped to pocket-shaped [30,32,33]. Additionally, most β-galactosidases from the Antarctic bacteria belonged to GH2 or GH42 [34,35,36,37]. However, in contrast to other β-galactosidases from Antarctic bacteria, GalA was not a cold-adapted enzyme, although its amino acid sequence had high homology with that from *Psychromonas* sp., and its catalytic activity was similar to that of mesophilic bacteria. Therefore, GalA was considered an interesting enzyme for further investigation.

### 2.2. Expression, Purification, and Characterization of GalA

**Figure 3:**
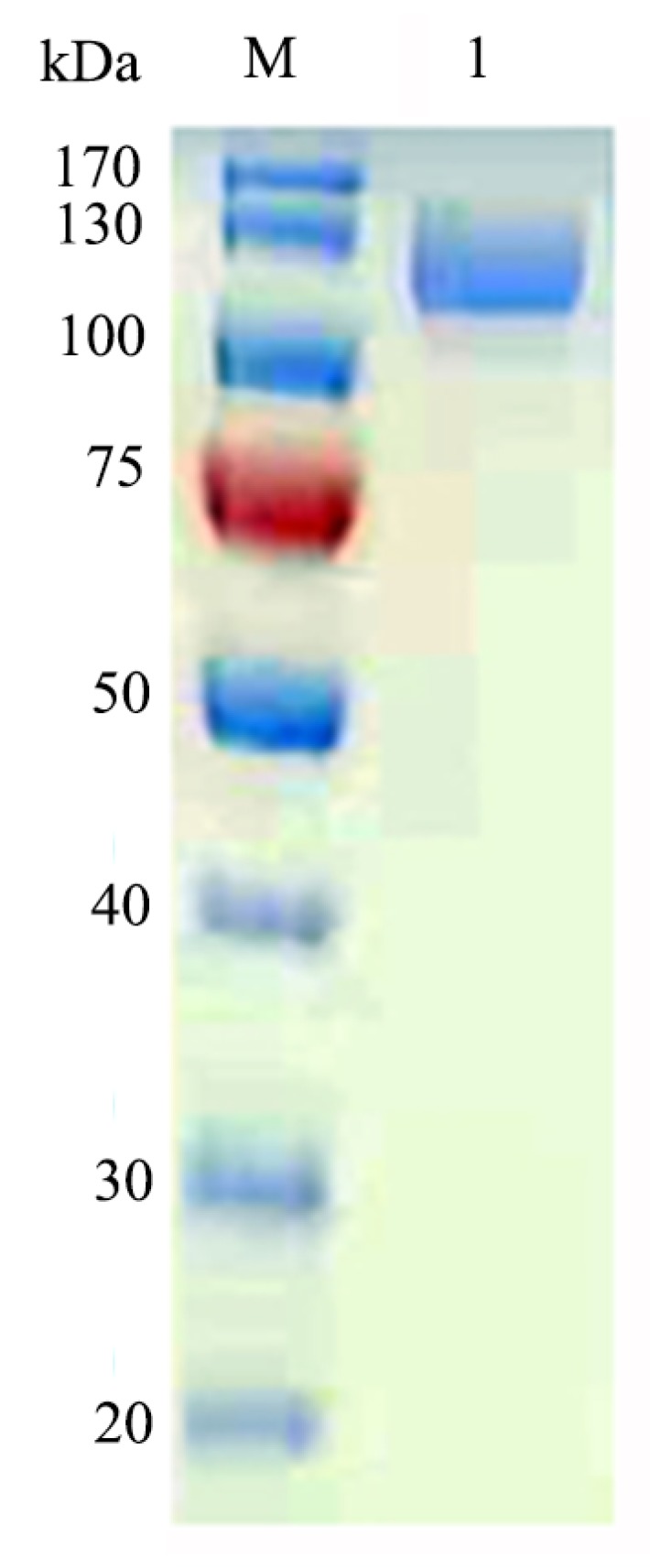
SDS-PAGE analysis of the recombinant GalA. *Lane* M, protein marker; *Lane* 1, the purified GalA.

The *E. coli* BL21-pET28a-GalA strain was grown in LB broth and induced by IPTG. The crude enzymes were harvested by centrifugation and purified by a Ni-Sepharose affinity column. The yield of GalA reached to 127 mg/L. After purification, the specific activity of GalA was 237.6 U/mg with 2-Nitrophenyl-β-d-galactopyranoside (ONPG) as substrate. SDS-PAGE analysis was used to determine the Mw of the purified enzyme. As shown in Figure 3, only a single clear band on the gel with an approximate Mw of 110 kDa was observed, which was corresponding to its theoretical Mw (117.4 kDa).

**Figure 4:**
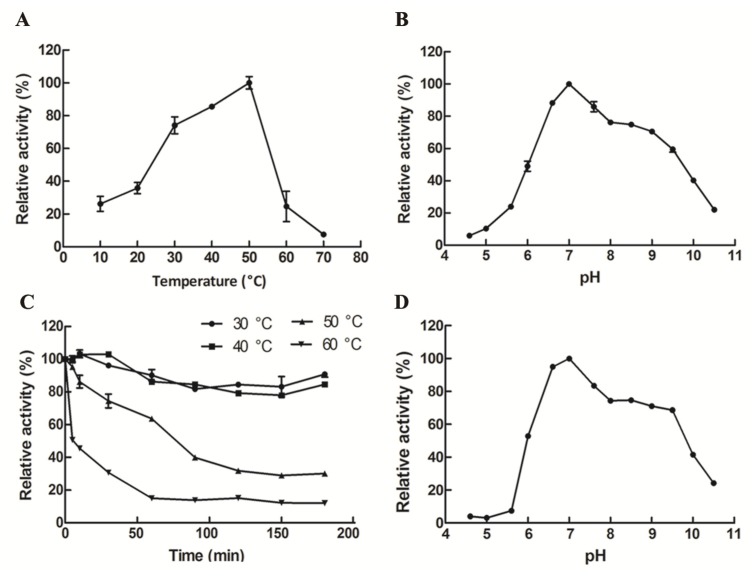
Effect of temperature and pH on GalA. (**A**) The optimal temperature of GalA; (**B**) the optimal pH of GalA; (**C**) the thermal-stability of GalA; and (**D**) the pH stability of GalA.

The optimum temperature for recombinant GalA was 50 °C (Figure 4A). Importantly, GalA maintained relatively high activity (>73.6%) at the temperature range of 30–50 °C. As shown in Figure 4C, more than 80% of the enzymatic activity remained after incubation below 40 °C. Meanwhile, the half-life time (t_1/2_) of GalA at 50 °C and 60 °C is 76.5 min and 27.6 min, respectively. The optimum pH (6.6–7.0) of GalA that is appropriate to the dairy environment will greatly facilitate its further application. Herein, the optimal reaction pH of GalA was 7.0 (Figure 4B). Meanwhile, GalA was stable in a pH range between 6.6 and 9.6, especially between 6.6 and 7.0 (Figure 4D), which will greatly enhance its application potential.

Reactions were carried out to test the impact of metal ions, EDTA, and SDS on the activity of GalA. As shown in Table 1, in general, enzymatic activity was activated by Mn^2+^, Mg^2+^, and Fe^3+^, and inhibited by Na^+^, K^+^, Li^+^, NH_4_^+^, Zn^2+^, Cu^2+^, Fe^2+^, Ba^2+^, Co^2+^, Ca^2+^, Al^3+^, and EDTA, as well as SDS (Table 1).

The substrate specificity of GalA was determined using five different substrates. As shown in Table 2, the enzyme was preferred ONPG as its substrate. Meanwhile, GalA showed 14.6% relative activity towards 4-Nitrophenyl-β-d-galactopyranoside (PNPG) and no activity to other substrates.

### 2.3. Docking Analysis of GalA

**Figure 5:**
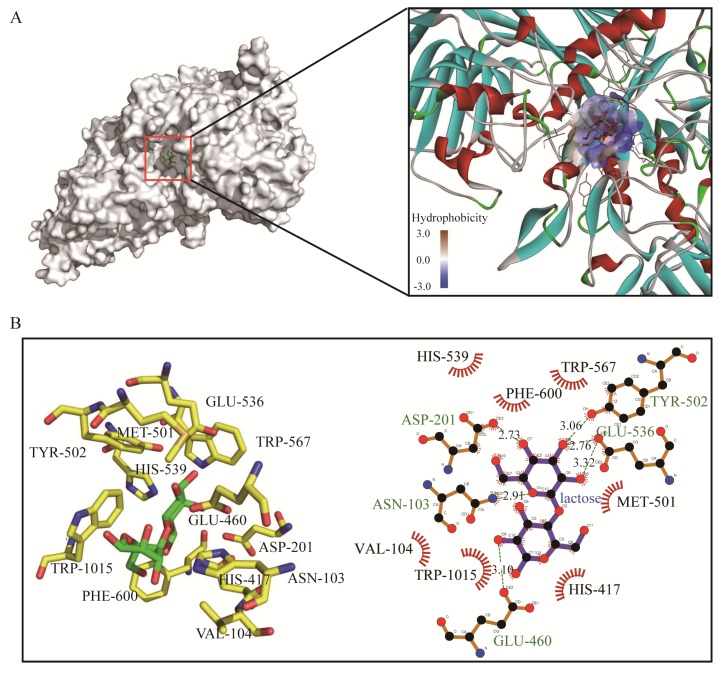
Molecular docking of GalA with lactose. (**A**) Overall and hydrophobicity analysis. (**B**) Schematic representation showing enzyme/substrate interactions of GalA. Hydrogen bonds are dotted lines and hydrophobic interactions are represented by "arcs".

The three-dimensional (3D) model of the GalA was constructed based on the homologues structure of β-galactosidases from *E. coli* (PDB ID: 6CVM) with an identity of 49.3%, and lactose was docked into the GalA. The docking parameters are shown in Table 3. Molecular docking indicates that lactose binds to a semi-open pocket on the catalytic domain of the protein, which has a good fit and geometric complementarity. The pocket is highly hydrophilic due to its proximity to the solvent environment. Lactose itself is also a hydrophilic molecule, thus facilitating its binding (Figure 5A). The β-galactosidase catalyzed the hydrolysis of lactose and transglycosylation to form GOS. The recombinant GalA exhibited high catalytic activity (237.6 U/mg) and efficiently degraded the lactose in milk. To identify the key residues for substrate recognition, the protein–substrate interactions were analyzed in ligplot. As shown in Figure 5B, the residues ASN-103, ASP-201, GLU-460, TYR-502, and GLU-536 form hydrogen bonds with lactose, respectively. Meanwhile, there are seven amino acids (VAL-104, HIS-417, MET-501, HIS-539, TRP-567, PHE-600, and TRP-1015) forming hydrophobic interaction towards lactose. These results suggested that GalA has a strong binding ability towards lactose to facilitate hydrolysis.

### 2.4. Hydrolysis of Lactose in Milk

**Figure 6:**
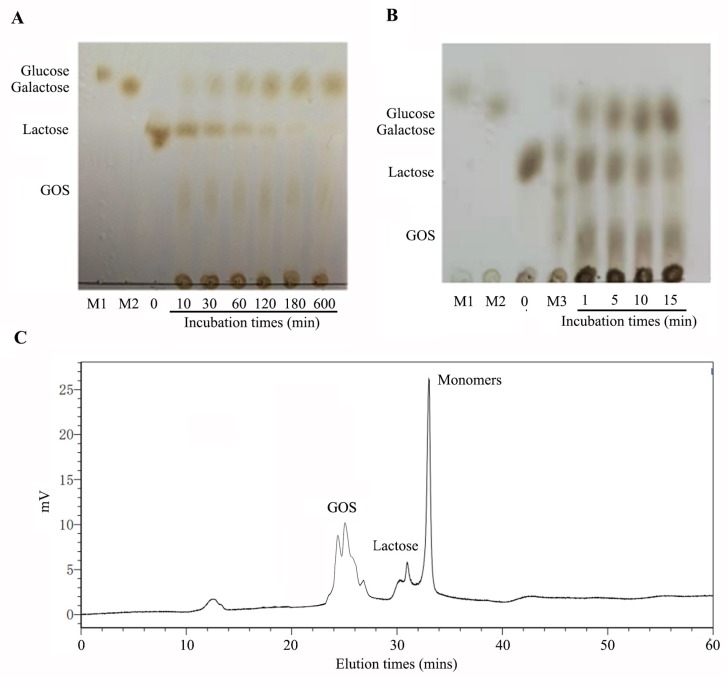
TLC and HPLC analysis of lactose and milk degrading by GalA. (**A**) TLC analysis of lactose degrading by GalA; (**B**) TLC analysis of milk degrading by GalA; and (**C**) HPLC analysis of GOS producing by GalA incubated for 15 min in milk.

β-Galactosidases catalyze the lactose hydrolysis and transgalactosylation to form GOS. To determine the lactose degrading and transgalactosylation activity of GalA, the products of GalA at different reaction times were analyzed by thin layer chromatography (TLC) (Figure 6A). As the reaction processing, lactose can be degraded into monomers. Meanwhile, GOS can appear clearly on the TLC plate. Finally, when the reaction time is extended to 600 min, lactose was completely hydrolyzed by GalA. There results indicated that GalA could efficiently play lactose degrading and transgalactosylation activity. Thus far, β-galactosidases are commercially used for the hydrolysis of lactose in dairy products, such as milk and cheese whey. In this study, we also determined the lactose-degrading effect in milk. As shown in Figure 6B, lactose in milk can be degraded fast. Meanwhile, GOS was formed along with the reaction processing. The reaction product at 15 min was further analyzed by size-exclusion high performance liquid chromatography (SE-HPLC) using a Superdex peptide 10/300^TM^ column. As shown in Figure 6C, 90.6% of the lactose was hydrolyzed and the GOS yield was 30.9%.

More than 90% of the population in Eastern Asia exhibit varying degrees of lactose intolerance. β-Galactosidases catalyze the lactose hydrolysis and has been commercially used in the dairy industry. Although various β-galactosidases have been cloned and characterized, only a few of them could be used for commercial applications. Thus far, the major industrial β-galactosidases are obtained from the *Aspergillus* and *Kluyveromyces* genera, which exhibit many excellent properties [38,39], such as an optimal pH (6.6–7.0) suitable to the dairy environment. This study indicated that GalA had optimal activity at pH 7.0 and was stable at pH 6.6–7.0, which corresponded with its application to milk. Thermostability is another important limitation of an enzyme application. A thermostable β-galactosidase gene *bgaB* was over-expressed in *B. subtilis* WB600 [40]. This enzyme was considered as one of the most thermally stable β-galactosidase, with half-life times at 65 and 70 °C of 50 and 9 h, respectively. In lactose hydrolysis, the maximum lactose degrading rate was 100% when using recombinant BgaB at 65 °C for 120 min. In this study, GalA could keep stable under 40 °C, which makes maintaining enzyme activity at normal temperatures possible (Figure 4C). Even GalA does not have the same thermal stability as BgaB, 90.6% of the lactose in milk could hydrolysis within 15 min at 40 °C. Comparing with other β-galactosidases from various bacteria, the cold-adapted β-galactosidase from *P. haloplanktis,* which hydrolyzed 26% of the lactose in milk at 25 °C under 30 min, this enzyme showed a lower apparent optimum temperature of activity and exhibited a weaker thermal stability [41]. Another β-galactosidase from *Lactobacillus pentosus* shows an optimal pH at 7.5–8.0 and obtain a maximum yield of 31% GOS of total sugars at 78% lactose conversion [42]. A recombinant β-galactosidase from *Lactobacillus plantarum* WCFS1, had a yield of 41% (w/w) of total sugars at 85% lactose conversion [43]. These comparisons indicated that GalA is suitable for lactose treatment in the dairy industry. 

Another important feature of β-galactosidase is the transgalactosylation reaction to form GOS. Being a nondigestible prebiotic, GOS could assist in improving the effect of intestinal flora on human health and stimulating the growth of beneficial bacteria such as bifidobacteria and lactobacilli [1]. The growth of probiotics can improve immunity and prevent the growth of pathogenic bacteria [3]. Meanwhile, GOS showed various benefits for the human health, such as decreasing the pH of human feces, preventing cariogenicity, and reducing serum cholesterol levels [1,2]. However, the transgalactosylation activity of β-galactosidases are different. The range of oligosaccharide varies between 1% and 45% in GOS that further depends on the total amount of saccharides and on the enzyme source of the enzyme [1]. Recently, many studies aimed to find new β-galactosidases to achieve higher GOS content and quality. Currently considered β-galactosidases from *Bacillus* species were investigated to obtain high GOS production [1]. β-galactosidase from *B. longum* BCRC 15708 produced GOSs (mainly trisaccharides) [44]. Recombinant β-galactosidase from *B. infantis* in *Pichia pastori* was widely used for commercial production of GOS. The conversion rates of transgalactosylation were about 25.2%, and the maximum GOS yield was 40.6% [45,46]. In this study, GOS showed a high conversion rate (30.9%), which will greatly improve its application potential.

## 3. Material and Methods

### 3.1. Bacterial Strains and Cell Culture Conditions

The seawater samples were collected from the surface layer of the Drake Passage near the Great Wall Station in the Antarctic. Bacteria in the seawater sample were collected by filtering 5 L of seawater onto 0.2 μm pore size filters (Pall, Lane Cove, Australia) and then serially diluted to 10^-6^ and placed on Zobell 2216E medium (BD Difco, Franklin Lakes, NJ, USA). After incubation at 15 °C and 25 °C for 7 days, more than 180 strains were isolated, which were inoculated onto selective medium (1% peptone, 1% yeast extract, 0.5% lactose, 2% NaCl, 0.004% X-gal, 1.5% agar, pH 7.0) for screening the β-galactosidase producer. One of strains with high activity was identified as *Alteromonas* sp. ANT48 based on the full-length sequence of its 16S rDNA and sequence alignment using the BLASTn algorithm program and MEGA 6.0. *Alteromonas* sp. ANT48 was grown in Zobell 2216E medium and was kept in 40% glycerol at −80 °C.

### 3.2. Sequence Analysis

The sequences of two primers, gal3—CTAGCTAGCATGCTTAATGTTGCGCAAGT and gal4—CCGCTCGAGTTATATTGCTTCAAAAAGG, used for cloning of the *Alteromonas* sp. ANT48 gene *GalA*, were designed based on the sequence of the β-galactosidase gene from *Alteromonas* sp. strain R10SW13 (Genbank accession number WP120962826). BLASTn and BLASTp (NCBI) were used to investigate the sequence homologies and protein sequence alignments, CLUSTALX program was used to perform multiple alignment analysis of GalA, and the MEGA 7.0 software was used to construct the evolutionary tree. Additionally, the ORF finder in NCBI (https://www.ncbi.nlm.nih.gov/orffinder/) was used to identify the open reading frame and the pI/Mw Tool (https://web.expasy.org/compute_pi/) was used to calculate the theoretical pI and Mw of GalA. Comparison of the results obtained using three databases, including CAZy (http://www.cazy.org/), CDD in NCBI (http://www.ncni.nlm.nih.gov/cdd), and Pfam database (https://pfam.xfam.org/), revealed the conserved and catalytic domains of GalA.

### 3.3. Overexpression and Purification of Recombinant GalA

For over-expression of the β-1,3 galactosidase GalA, expression primers (PgalA-EF and PgalA-ER) were used to amplify the genome of the Antarctic seawater bacterium *Alteromonas* sp. ANT48. The signal peptide and stop codons were removed in the designation of expression primers. Afterwards, the obtained *galA* gene was ligated into the vector pET-28a with the recognition sites *Nco* I and *Xho* I. Finally, the recombinant plasmid pET-28a-galA was translated into *E. coli* BL21 (DE3). The recombinant strains grew in TB broth with 30 μg/mL kanamycin at 37 °C. When the OD_600_ of recombinant *E. coli* BL21 (DE3)-pET-28a-galA reached 0.6−0.8, 0.1 mM isopropyl β-D-thiogalactoside (IPTG) was added into the growth medium to induce the expression of proteins. The incubation was continued for 36 h at 20 °C with shaking at 200 rpm. The recombinant strains were harvested by centrifugation and suspended at 50 mM phosphate buffer, pH 7.6. Then, the solution was disrupted by sonication at ice condition. The total purification protocol was performed using the AKTA150 FPLC system (GE Healthcare, Madison, WI, USA). After removing the cell debris, the supernatant was collected and loaded onto the Ni-Sepharose affinity column (5 mL His-Trap^TM^ High Performance, GE Healthcare, Madison, WI, USA), which was equilibrated previously. The impurity protein was removed using the washing buffer (20 mM imidazole, 20 mM phosphate buffer, 500 mM NaCl, pH 7.6). The active fractions were collected using the eluting buffer (150 mM imidazole, 20 mM phosphate buffer, 500 mM NaCl, pH 7.6). To analyze the Mw and purity of the purified GalA, 10% SDS-PAGE was used, and the PageRuler Prest Protein Ladder (Thermo Scientific, Waltham, MA, USA) was used as a protein standard marker.

### 3.4. Characterization of Recombinant GalA

The effect of pH and temperature on the purified GalA was analyzed as follows. The optimal reaction temperature of GalA was determined at the temperatures, ranging from 0 to 70 °C in 20 mM phosphate buffer, pH 7.2. The optimal reaction pH of GalA was determined in 50 mM citrate buffer (pH 3.6–5.6), 50 mM phosphate buffer (pH 5.8–7.4), 50 mM Tris-HCl buffer (pH 6.4–7.8) and 50 mM Gly-NaOH buffer (pH 8.0–9.6), respectively. To determine the thermostability of the enzyme, GalA was pre-incubated at 30 °C, 40 °C, 50 °C, or 60 °C for various times. Then, the residual activity of the enzyme was determined at its optimal temperature and pH. To analysis its pH stability, the enzyme solution was pre-incubated in various pH buffers (pH 3.4–9.6) at 4 °C for 12 h. Then, the residual activity was measured under normal assay conditions. The effects of different metal ions and detergents on the enzyme activity of GalA were measured in the presence of various compounds (1 mM) in the substrate solution. The substrate specificity of the GalA was determined using 5 mM of five different substrates, including 4-Nitrophenyl-ɑ-d-galactopyranoside, 4-Nitrophenyl-β-d-glucopyranoside ONPG, PNPG, or 4-Nitrophenyl-β-d-xylopyranoside in 50 mM sodium phosphate buffer (pH 7.0) under the optimum conditions.

### 3.5. Docking Analysis

The 3D-structure of β-galactosidases GalA was built by the method of homology modeling using the Modeller 9.18 package (https://salilab.org/modeller/). The crystalline structure of β-galactosidases from *E. coli* (PDB ID: 6CVM) was chosen as the template. A total of 100 structures were built, and the structure with the best Discrete Optimized Protein Energy (DOPE) score was chosen for further docking analysis. The ligand (lactose) was drawn by ChemDraw V12.0. Then, the AutodockTool (The Scripps Research Institute, San Diego, CA, USA) was used for docking. The tool identifies novel binders by predicting their binding modes and affinities. Pymol was used to visualize the docking solutions and construct the graphical presentations and the figure illustrations. The conformation of lowest affinity value was chosen for further analysis in ligplot.

### 3.6. Activity Assay of β-Galactosidase 

The substrate, ONPG (Sigma Alddrich, St. Louis, MO, USA), was used for the β-galactosidase activity assay. Enzymatic activity was performed using 100 µL enzyme and 900 µL substrate (0.3% (w/v) in phosphate buffer, pH 7.0) at 50 °C for 10 min. Then, 200 µL of 1 M Na_2_CO_3_ was added into the reaction mixture to end reaction. Then the reaction mixture was determined at OD 420 nm according to the ONP standard curve. One unit (U) of β-galactosidase was defined as the amount of enzyme required for the liberation of 1 µmol ONP per minute under the assay conditions [47].

### 3.7. Analysis of the Hydrolysis of Lactose and Galactooligosaccharides Production

The reaction products of GalA towards lactose were determined by TLC analysis. Briefly, 100 µL of purified GalA (100 U) was mixed into 900 µL lactose substrate (5 mg/mL) and incubated for various times (0 and 30 min, and 1, 2, 6, and 12 h). Then, the reaction product was analyzed using TLC analysis on a HPTLC plate (Merck, Darmstadt, Germany) that was developed with a mixture n-butanol/formic acid/water (5:3:2, by vol) reagents. After drying and coloring at 80 °C for 30 min, the TLC plate was visualized with a solution of 90% (v/v) ethanol and 10% (v/v) sulfuric acid reagent. The hydrolysis of lactose in milk was determined by incubating 100 μL purified GalA in 900 μL of commercial skim milk (Inner Mongolia Yili Industrial Group Co. Ltd., Hohhot, China) at 50 °C for different times (1, 5, 10, and 15 min) and then boiling the reaction mixture to end the reaction. The supernatant was analyzed by TLC analysis. To further analysis the ratio of GOS in the milk degrading of GalA, the reaction product at 15 min was analyzed by HPLC with a superdex peptide 10/300 ^TM^ column. The reaction products of GalA were analyzed by SE-HPLC on the ÄKTA Avant 150 platform (GE Health, Chicago, IL, USA) using a Superdex peptide 10/300^TM^ column (GE Health) equipped with a refractive index detector. The mobile phase was 0.2 M ammonium bicarbonate. The column pressure was limited to 1.5 MPa and the flow rate was 0.5 mL/min. 

### 3.8. Nucleotide Sequence Accession Numbers

The 16S rRNA gene sequence of Antarctic seawater bacterium *Alteromonas* sp. ANT48 and β-galactosidase gene (*galA*) were deposited in GenBank under the accession numbers MN332242 and MN337572, respectively.

## 4. Conclusions

In this study, we cloned and characterized a new GH2 β-galactosidase, GalA, from the Antarctic bacterium *Alteromonas* sp. ANT48. Our study demonstrated that the combined properties of GalA, being an optimal pH at 7.0 and stable at a pH range of 6.6–7.0, efficient lactose degrading ability, and high GOS yield make it an excellent candidate for further research and adaptation for commercial use. Further analyses will focus on elucidating the molecular mechanism of GalA yielding GOS, along with the determination of its three-dimensional structure. This approach also provides a theoretical basis for the preparation of other marine glycoside hydrolase enzymes.

## Figures and Tables

**Table 1:** Effects of metal ions, EDTA and SDS on the activity of GalA.

Reagent Added	Concentration (mM)	Relative Activity (%)
None	-	100.0 ± 0.0
NaCl	10	54.9 ± 2.3
SDS	1	17.9 ± 3.7
EDTA	1	26.2 ± 3.9
Al_2_(SO_4_)_3_	1	21.7 ± 3.0
KCI	1	67.5 ± 0.9
CuSO_4_	1	8.1 ± 1.0
FeCI_2_	1	77.8 ± 4.2
(NH_4_)_2_SO_4_	1	93.7 ± 1.7
MnSO_4_	1	164.1 ± 1.4
Li_2_SO_4_	1	64.7 ± 2.0
ZnCI_2_	1	77.2 ± 2.1
BaCI_2_	1	26.9 ± 5.1
CoCI_2_	1	69.4 ± 2.2
MgCI_2_	1	149.0 ± 3.8
CaCI_2_	1	82.9 ± 2.2
FeCI_3_	1	142.7 ± 1.0

Notes: Activity without addition of chemicals was defined as 100%. Data are shown as means ± SD *(n* = 3).

**Table 2:** Substrate specificity of the recombinant GalA.

Substrate	Relative Activity (%) ^1^
2-Nitrophenyl-β-d-galactopyranoside (ONPG)	100 ± 7.9
4-Nitrophenyl-β-d-galactopyranoside (PNPG)	14.6 ± 3.1
4-Nitrophenyl-ɑ-d-galactopyranoside	0
4-Nitrophenyl-β-d-glucopyranoside	0
4-Nitrophenyl-β-d-xylopyranoside	0

^1^ Activity of ONPG substrate was defined as 100%. Data are shown as means ± SD (*n* = 3).

**Table 3:** Docking parameters in Autodock software.

Parameters	Value
X coordinate of the center	81.551
Y coordinate of the center	130.691
Z coordinate of the center	103.074
Size in the X dimension	24 Å
Size in the Y dimension	24 Å
Size in the Z dimension	24 Å
Maximum number of binding modes to generate	9
Maximum energy difference between the best	4 kcal/mol
